# Single-neuron RNA-Seq: technical feasibility and reproducibility

**DOI:** 10.3389/fgene.2012.00124

**Published:** 2012-07-06

**Authors:** Shenfeng Qiu, Shujun Luo, Oleg Evgrafov, Robin Li, Gary P. Schroth, Pat Levitt, James A. Knowles, Kai Wang

**Affiliations:** ^1^Zilkha Neurogenetic Institute, University of Southern CaliforniaLos Angeles, CA, USA; ^2^Department of Cell and Neurobiology, University of Southern CaliforniaLos Angeles, CA, USA; ^3^Illumina, Inc, HaywardCA, USA; ^4^Department of Psychiatry, University of Southern CaliforniaLos Angeles, CA, USA

**Keywords:** cell culture, electrophysiology, gene expression, neuron, RNA-Seq, transcriptome

## Abstract

Understanding brain function involves improved knowledge about how the genome specifies such a large diversity of neuronal types. Transcriptome analysis of single neurons has been previously described using gene expression microarrays. Using high-throughput transcriptome sequencing (RNA-Seq), we have developed a method to perform single-neuron RNA-Seq. Following electrophysiology recording from an individual neuron, total RNA was extracted by aspirating the cellular contents into a fine glass electrode tip. The mRNAs were reverse transcribed and amplified to construct a single-neuron cDNA library, and subsequently subjected to high-throughput sequencing. This approach was applied to both individual neurons cultured from embryonic mouse hippocampus, as well as neocortical neurons from live brain slices. We found that the average pairwise Spearman’s rank correlation coefficient of gene expression level expressed as RPKM (reads per kilobase of transcript per million mapped reads) was 0.51 between five cultured neuronal cells, whereas the same measure between three cortical layer 5 neurons *in situ* was 0.25. The data suggest that there may be greater heterogeneity of the cortical neurons, as compared to neurons *in vitro*. The results demonstrate the technical feasibility and reproducibility of RNA-Seq in capturing a part of the transcriptome landscape of single neurons, and confirmed that morphologically identical neurons, even from the same region, have distinct gene expression patterns.

## INTRODUCTION

Prior to the advent of high-throughput methods to measure the entire transcriptome, such as microarrays or next-generation sequencing, studies of gene expression and function in the brain were restricted to a relatively small number of genes (Luo and Geschwind, 2001; Zhang et al., 2002). Recently, whole transcriptome sequencing (RNA-Seq) has enabled the measurement of abundance of tens of thousands of RNA species in a given biological sample (Wang et al., 2009; Ozsolak and Milos, 2011). This new generation of high-throughput sequencing technology has delivered on its promise of sequencing DNA, cDNA, and RNA at unprecedented speed and accuracy, thereby providing an increasingly wide-ranging array of data sets that provide insight into biological and disease diversity (Schuster, 2008). Single-cell analysis represents one of the novel areas of application for high-throughput sequencing, which is particularly important for the study of tissues that have a high degree of intrinsic variation, such as the brain. To date, this technology has achieved success in tumor profiling to study somatic DNA mutations in clonal sub-populations: for example, Navin et al. (2011) applied single-nucleus DNA sequencing to investigate tumor population structure and evolution in human breast cancer cases. Single-cell RNA sequencing also can be used to study differences of individual cells with identical genomes, for example, in a pool of neurons from a single brain.

There is a strong rationale to perform single-neuron RNA expression analysis. The mammalian brain consists of billions of neurons that each typically exhibits over 100,000 macroconnections (Bota et al., 2003; Luo et al., 2008). Individual neurons can be characterized into distinct cell types based on morphology, electrophysiological characteristics, connections, and expressed molecular diversity. Such cell-specific information is diluted when pooling groups of neurons. Gene expression differences occurring in rare cell types may go undetected, because they contribute to only a small fraction of total tissue RNA. Moreover, gene expression may be regulated in opposing directions in different cell types, thereby appearing static in composite data. Cell type-specific transcriptomics will provide a more panoramic view of gene expression, and ultimately networks, rather than from a viewpoint dominated by the effects of single genes. Moreover, correlating gene expression data of individual neurons with single-cell phenotypic data has the potential to refine cell type definitions.

Transcriptome analyses of single neurons have been reported using mostly microarrays. Earlier pioneering studies of single-cell microarray analysis used a universal PCR amplification procedure, or two rounds of T7 RNA polymerase amplification. For example, Esumi et al. (2008) developed method for single-cell microarray analysis and applied them to gene-expression profiling of GABAergic neuron progenitors. Iscove et al. (2002) demonstrated that the representation is faithfully preserved in global cDNA amplified exponentially from sub-picogram quantities of mRNA. Kamme et al. (2003) examined expression profiling of CA1 neurons in the rat hippocampus using a combination of laser capture, T7 RNA amplification, and cDNA microarray analysis. Single GABAergic neuron progenitors from mouse neocortex were isolated by dissociation and aspiration of green fluorescent protein (GFP)-positive cells, then processed by Super SMART PCR and T7 RNA polymerase amplification. Similarly, Gustincich et al. (2004) combined SMART PCR and T7 RNA polymerase amplification to interrogate the transcriptome in single dopaminergic neurons of the retina in mice. Tietjen et al. (2003) also used similar techniques in single cells from dissociated tissues or collected from intact slices using laser capture. These studies demonstrated that microarray-based analysis of gene expression can be performed on single neuronal cells isolated from defined areas of the brain.

Compared with microarray methods, sequence-based transcriptome profiling has major advantages, such as extended linear range of detection, accuracy, binary low noise reading, and independence from a reference genome. Next-generation sequencing techniques have been applied recently to single-cell transcriptome analysis. For example, Tang et al. (2009), 2010b used single-cell transcriptome analysis based on RNA-Seq and applied the methods to analyze the developmental program of embryonic stem cells (Tang et al., 2010a). Recently, Eberwine and Bartfai (2011) have examined the single-cell transcriptome in hypothalamic warm sensitive neurons that control core body temperature and fever response. These authors assayed cDNA libraries from single neurons using Affymetrix gene expression arrays, and confirmed the frequency of specific cDNAs by Illumina sequencing. The importance of single-cell transcriptome analysis has been increasingly recognized recently, especially for tracing cell lineages and in diagnostic applications (Stahlberg et al., 2011; Tang et al., 2011). The feasibility and reproducibility of RNA-Seq in single neurons, however, has not been systematically studied, though a protocol to assay single neurons by qPCR was recently published (Citri et al., 2012). Furthermore, experimental variability related to extremely low levels of RNA, and bioinformatics analysis of the RNA-Seq data, can be technically demanding. Our study differs from previous studies in that we developed protocols specifically for assaying neurons, and our method can be directly coupled with electrophysiology studies on individual neurons, thus enabling the correlation of single-cell cellular phenotypes with single-cell expression phenotypes. Below we describe the experimental strategy and application that was designed to test the practicality of single-neuron RNA-Seq using equipment readily available in a typical neurophysiology laboratory. The study demonstrates the ability to generate RNA-Seq data with reasonable reproducibility from individual, electrophysiologically characterized neurons.

## MATERIALS AND METHODS

### COLLECTION OF SINGLE NEURON CONTENTS

To assess the technical feasibility of RNA-Seq for single-neuron transcriptome profiling, we started with cultured mouse embryonic hippocampal neurons. All procedures used C57Bl6 mice, and were approved by the Institutional Animal Care and Use Committee of the University of Southern California. Animal care and handling conformed to National Institutes of Health guidelines. Hippocampal neuronal cultures were prepared from embryonic day (e) 16–17 mice according to standard protocols (Qiu and Weeber, 2007). Briefly, hippocampi from fetal brains were collected and digested in Hank’s balance salt solution containing 0.5 mg/ml of papain at 37°C for 20 min. The hippocampi were then separated into a single-cell solution using aspiration with a glass micropipette. The cells were seeded on poly-D-lysine-coated 12-mm glass coverslips at a density of 10,000 cells/cm^2^ and grown in Neurobasal medium supplemented with 2% B27. Under these growth conditions, most neurons exhibit a glutamatergic phenotype.

At 12 days *in vitro* (DIV), a glass coverslip with cultured neuron was transferred into an electrophysiology recording chamber that was perfused by circulating artificial cerebrospinal fluid (ACSF, containing in mM: 126 NaCl, 2.5 KCl, 26 NaHCO_3_, 2 CaCl_2_, 2 MgCl_2_, 1.25 NaH_2_PO_4_, and 10 D-glucose)). It is critical that ACSF is freshly made with milliQ water and the perfusion system is flushed with ACSF at least twice to maintain a “clean” surrounding for neurons. Neurons were visualized on an Olympus BX51WI microscope equipped with a ×60 water immersion lens and infrared differential interference contrast (DIC) optics, and a focusing step motor that is driven by a Sutter MP285 motion controller. A patch electrode pulled from borosilicate glass was filled with the recording solution: (in mM: 126 K-gluconate, 4 KCl, 10 HEPES, 4 ATP, 0.3 GTP, 10 mm phosphocreatine, and 0.4 Unit/μl RNaseOUT). This patch electrode had an electrical resistance of 2.5–3 MΩ in the open ACSF bath. The electrode was mounted on a micromanipulator (Sutter MP 285) and was placed over the target neuron under visual guidance. Only neurons with a pyramidal-shaped soma were selected for analysis to avoid potential interneurons. The electrode was sealed on the neuron by applying negative pressure. Whole cell configuration was obtained by breaking into the neuron following a gigaOhm seal. Neurons were then voltage clamped at their resting membrane potential with a Multiclamp 700B amplifier controlled by pClamp 10.2 software. Spontaneous neuronal activity was acquired for 30 s followed by a series of current step injections to verify that the neurons were healthy. In cases where there is a large (>100 pA) holding current and truncated action potential firing in response to current injection (neurons being “leaky”), neuronal content was not collected for analysis. Following the brief recording, stronger negative pressure was applied to draw all the soma contents into the electrode. The electrode was rapidly retracted from the bath, and the contents expelled into a thin-wall PCR tube (mounted on a manual manipulator) with 3.5 μl lysis buffer. This lysis buffer was made from nuclease free water with 2% Triton X-100, and 5% RNaseOUT (Invitrogen). The collected single cell contents were then immediately frozen on dry ice and stored at -80°C until further processing.

To obtain the cell contents of live single neurons *in situ* from brain tissue, sagittal sections were prepared from postnatal day (P) 21–28 neocortex, similar to that described previously (Qiu et al., 2010, 2011). Briefly, mice were deeply anesthetized by isoflurane and quickly decapitated. Brains were dissected and sectioned on a vibratome in ice-cold, carbogenated choline solution containing (in mM: 110 choline chloride, 25 NaHCO_3_, 2.5 KCl, 1.25 NaH_2_PO_4_, 0.5 CaCl_2_, 7 MgSO_4_, 25 D-glucose, 11.6 sodium ascorbate, and 3.1 sodium pyruvate). Sagittal slices (350 μm thick) of the right hemisphere containing prefrontal cortex were cut. Slices were then incubated in ACSF for 30 min at 35°C and maintained at 22°C. A single slice was then transferred to the recording chamber with clean ACSF perfusate (see above). Under visual guidance, layer 5 pyramidal neurons in the anterior frontal cortex were targeted by a patch electrode. The patch electrode (3–3.5 MΩ) was filled with the same recording solution as described above. Prior to entering the ACSF, positive pressure in the electrode is applied so that the internal solution is flowing out during the whole process to avoid contamination of the intracellular solution by brain tissues. After the electrode approached the neuron under visual guidance, negative pressure is applied by mouth suction to form a gigaOhm seal. The cell membrane was ruptured by stronger, pulsate suction so that a continuity is formed between the electrode solution and neuronal content. The neurons are then tested for their membrane properties (membrane resistance, capacitance, time constant, resting membrane potential). Current steps were then injected into the neurons to test other electrophysiological properties (action potential threshold, spike frequency adaption). To aspirate the neuronal contents, strong negative pressure was applied with a syringe, until the soma was completely extracted into the electrode. The complete aspiration of soma content into the patch electrode is visualized under DIC optics and by focusing through various Z plane levels. The neuronal content was collected into a thin-walled PCR tube containing 3.5 μl lysis buffer, flash frozen on dry ice, and stored at -80°C.

### RNA-Seq LIBRARY PREPARATION AND DATA GENERATION

RNA-Seq library was generated following manufacturers recommended protocols (SMARTer^™^ Ultra Low input RNA Kit, Clontech Cat. 634935 and Illumina Paired-end DNA sample preparation kit Cat. PE-102-1001). The SMARTer kit can assay 100 pg of input RNA (well suited for single-cell analysis), and allows the efficient incorporation of known sequences at both ends of cDNA during first-strand synthesis in one step, without the adaptor ligation step. Briefly, the PCR tubes containing the cytoplasmic content of a single cell in 3.5 μl of reaction buffer were thawed on ice, and all the following steps were carried out in a laminar flow hood to reduce the possibility of contamination from environmental DNA sources. First-strand cDNA synthesis was initiated by adding 1 μl of 3′ SMART CDS Primer II A (12 μM) at 72°C for 3 min, and then 5.5 μl Mastermix containing SMARTScribe^™^ Reverse Transcriptase (100 U) was added and the reaction was incubated at 42°C for 90 min, followed by inactivation at 70°C for 10 min. First-strand cDNA was isolated using SPRI Ampure Beads, and then double-strand cDNA were generated by long distance PCR for 18 cycles. Double-stranded DNA (dsDNA) was purified using SPRI AMPure beads, and the quality was assessed using an Agilent 2100 BioAnalyzer. The double-stranded cDNA fragments were sheared by Covaris, then end-repaired with a combination of T4 DNA polymerase, Klenow fragment polymerase, and poly nucleotide kinase to ensure blunted ends, and then adenylated with a single A-base at the 3′-end of the fragment by the Klenow 3′-5′ exo-enzyme. The tail was ligated with the Illumina adapters by T4 DNA ligase. The adapter-ligated sample was size selected by electrophoresis, and amplified by PCR using primers that only anneal to adapter-ligated fragments. The adapter sequences were annealed to the primers on the Illumina flow cell during bridge PCR in Illumina Genome Analyzer IIx sequencer, which generated the clusters necessary to view fluorescence during the sequencing-by-synthesis process.

### RNA-Seq DATA ANALYSIS

The FASTQ file and associated base quality scores were generated by factory software associated with the Genome Analyzer IIx sequencer. We used TopHat (Trapnell et al., 2009) version 1.2.0 for aligning the Illumina reads against the reference mouse genome (NCBI build M37) with default parameters. We used Cufflinks (Trapnell et al., 2010) version 1.0.3 to summarize the gene expression values as RPKM (reads per kilobase of transcript per million mapped reads) measures using the – compatible-hits-norm argument, so that only reference transcripts are counted toward the number of mapped hits used in the RPKM denominator. We also used the “-G” argument, which calculates the gene expression levels for all known/annotated transcripts. For the annotated transcript library, we used the latest ENSEMBL (Birney et al., 2004) GTF file, downloaded from ftp://ftp.ensembl.org/pub/release-62/gtf/mus_musculus/ after filtering to remove all mitochondrial and ribosomal transcripts after alignment.

## RESULTS AND DISCUSSION

### TECHNICAL OVERVIEW OF SINGLE-NEURON RNA-Seq

The analysis of each single-cell transcriptome consists of several independent steps. First, the cell type of interest is identified; this can often be done in neurons through genetic labeling or by characterizing the electrophysiology properties. In many cases, cell-specific promoters can be used to drive the expression of a marker gene such as GFP. Second, RNA from the targeted neuron must be extricated from the surrounding cells and tissues. For instance, this can be done by aspirating the cellular contents into a patch clamp recording electrode under visual guidance. Alternatively, GFP-positive cells can be selected through fluorescence activated cell sorting, or by laser capture microdissection in fixed brain tissues (Lin et al., 2007). Third, because the resulting RNA is typically very low in abundance, it must be amplified and converted to cDNA before being subjected to sequencing or microarray analysis.

**FIGURE 1:**
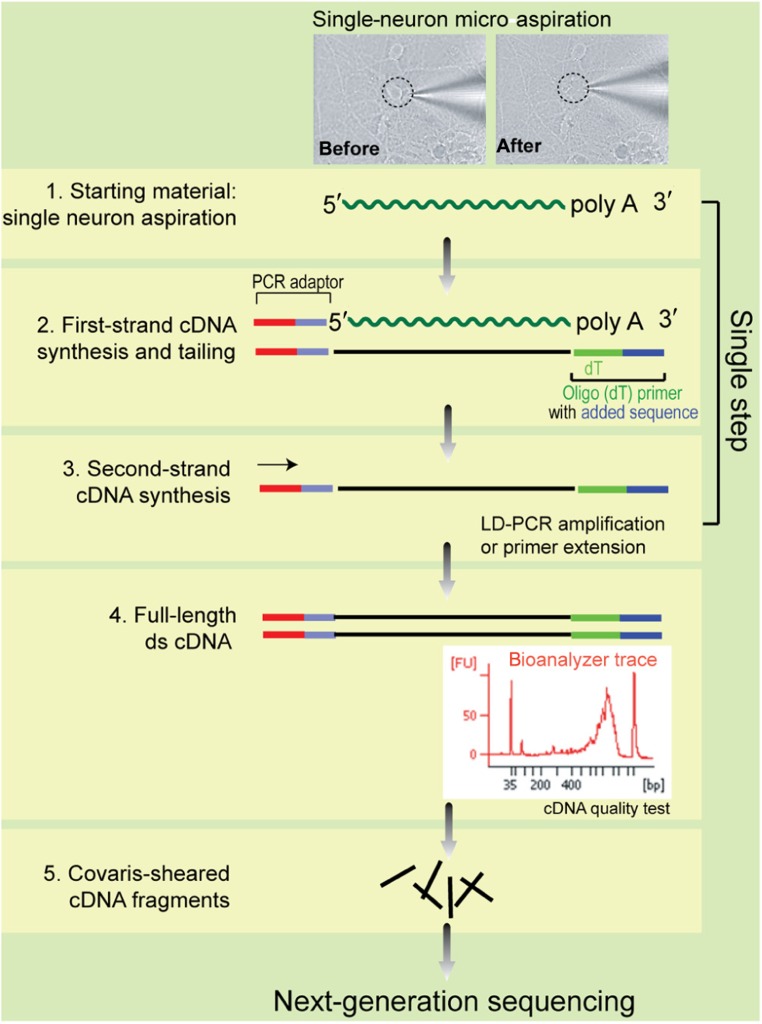
**Experimental design of the single-neuron RNA-Seq method reported in this study**.

To demonstrate the feasibility of single-neuron RNA-Seq analysis, we assayed three layer 5 pyramidal neurons from the same brain slice that exhibited typical electrophysiological properties, as well as five randomly selected pyramidal cells from hippocampal neuronal cell culture at 12 DIV (**Table 1**). To harvest cellular contents from live neurons in culture or in brain slices, we used a “direct aspiration” approach that does not require specific instrumentation or labeling by GFP. Double-stranded cDNA was generated from single cell content, and used in standard Illumina library construction and next-generation sequencing with an Illumina Genome Analyzer IIx (**Figure 1**). On average, ~22 million 50-bp reads were generated from each neuron. The sequence data was converted to FASTQ files, aligned against the mouse genome build NCBI M37 using TopHat software, and then analyzed by the Cufflinks program to quantify the expression levels for each transcript and gene.

**TABLE 1:** A list of samples used in our RNA-Seq experiment. The live neurons were retrieved from the same brain slice with normal electrophysiological properties.

Sample	ID	Description
HCT20466	Neuron 1	Living neuron from brain slice
HCT20468	Neuron 2	Living neuron from brain slice
HCT20469	Neuron 3	Living neuron from brain slice
HCT20470	4-neuron pool	A pool of 4 neurons from brain slice
HCT20575	Cell 1	Single neuronal cell from DIV12 culture, #18
HCT20576	Cell 2	Single neuronal cell from DIV12 culture, #19
HCT20577	Cell 3	Single neuronal cell from DIV12 culture, #20
HCT20578	Cell 4	Single neuronal cell from DIV12 culture, #21
HCT20579	Cell 5	Single neuronal cell from DIV12 culture, #22
HCT20580	Cell 6	Single neuronal cell from DIV12 culture, #24

### QUALITY CONTROL OF SINGLE-NEURON RNA-Seq

In conventional RNA-Seq experiments, the total quantity of RNA samples is typically sufficiently large (>100 ng), so that contamination of foreign RNA is not a major concern. However, in our experiments, given the limited amount of starting materials(typically around ~10 pg of total RNA), careful sample handling techniques become extremely important to reduce the possibility of contamination from multiple sources. In fact, our first set of experiments failed in that the vast majority of reads cannot be aligned and were subsequently traced back to pollen DNA, as the experiments were performed on an open lab bench during spring time in Los Angeles. It is therefore recommended that the retrieval of cell lysate and library preparation be performed under a laminar flow hood with extra caution to avoid any possible contamination. We found two critical things to reduce RNA contamination and degradation: (1) A good DIC optics will reveal clear neuronal boundary from surrounding cells and tissues. In combination with a continuous adjust of Z focus plane and a strong, pulsate suction using a glass syringe (glass plunger lubricated with water), we were able to minimize the suction of surrounding tissues/cell processes into the electrode. Nonetheless, possibility still exists that a small fraction of surrounding tissues and/or incomplete suction of neuronal content could contribute to the heterogeneity in reads (and low Spearman rank). (2) We have optimized the RnaseOUT concentrations in the electrode internal/harvesting solution (0.4 unit/μl or 1%) and the reaction buffer (5%), while other concentrations seemed to result in significant RNA degradation. It should be noted that microaspiration is incapable to capture neuronal contents in distal compartments, such as axons and dendrites, which may contain a small quantity of mRNA (Eberwine et al., 2001; Batish et al., 2012). This caveat notwithstanding, single-neuron RNA-Seq should faithfully capture the majority of transcriptome. A major advantage of this technique is that it can be done in live neurons in brain slices after electrophysiology recording, therefore offers valuable insights on how neuronal gene network determines each neuronal types (for example, neural stem cells differentiation, developmental specification of major neuronal types, such as excitatory projection neurons vs. local inhibitory neurons). Another advantage is that it may be possible to further develop aspiration-based techniques to determine the 3D organization of tissues such as the brain: we can envision a microfabricated array of thousands of micropipettes, under computer control, advancing through tissue, stopping, and aspirating once a cell is detected and then continuing to advance. Data from such an array would then enable 3D reconstruction of the tissue transcriptome. It is also conceivable that future RNA-Seq through the use of laser capture microdissection or other higher throughput microdissection devices may be a viable alternative to the single electrode aspiration approach.

### REPRODUCIBILITY OF RNA QUANTIFICATION

**FIGURE 2:**
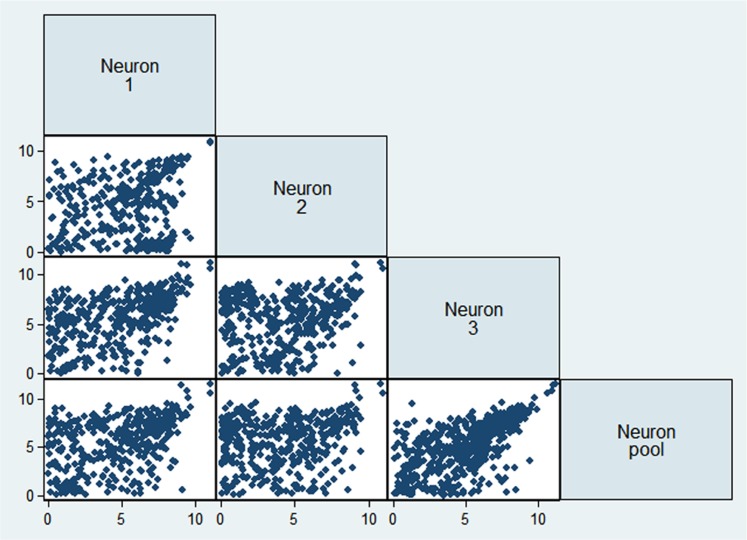
**Comparison of gene expression values (as logarithm of RPKM) from three single neurons and the 4-neuronal pool**.

**FIGURE 3:**
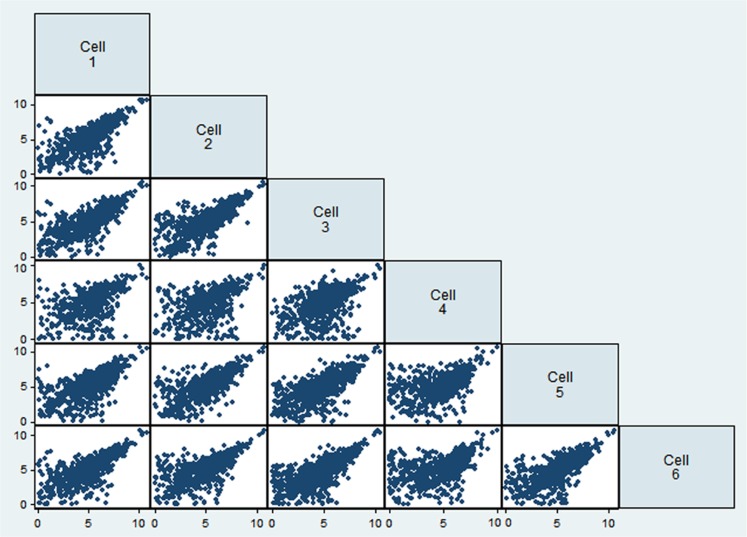
**Comparison of gene expression values (as logarithm of RPKM) for six single neuronal cells from cell culture**.

**FIGURE 4:**
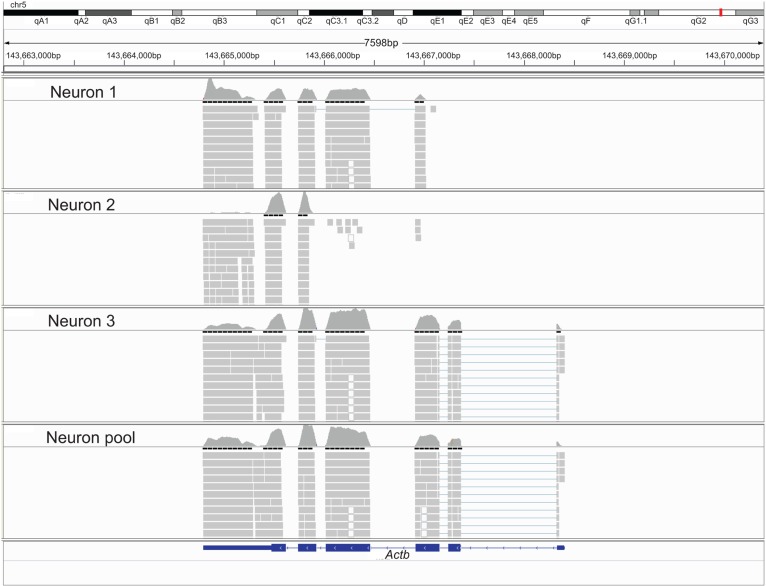
**A Integrative Genomics Viewer screen shot of the *Actb* locus, showing the coverage levels and alignments locations for three single live neurons and the 4-neuron pool from the same brain slice**.

To evaluate the reproducibility of RNA quantification, we compared gene expression levels (expressed as RPKM) of three individual and one pool of four neighboring mouse layer 5 pyramidal neurons from slices. The correlation of gene expression between single and pooled neurons was compared (**Tables 2 and 3, Figure 2**). Given the skewed distribution of RPKM values, Spearman’s rank correlation was used, rather than Pearson’s correlation. On average, the pairwise rank correlation coefficients of the RPKM values between individual pyramidal neurons *in situ* was 0.25, yet the same measure between each individual neuron and a 4-neuron pool was 0.35. In comparison, we also examined six single cultured mouse embryonic hippocampal neurons harvested at 12 DIV. The average pairwise Spearman rank correlation coefficients was 0.51 for six individual neurons grown in cell culture (**Figure 3**). Although we did not perform technical replicates in RNA-Seq, our previous experience showed that correlations between technical replicates are typically >0.95, so that noises from the sequencing run per se are unlikely to have major influence. Our results suggest that the lower correlation value for different neurons in brain slices may reflect cell type heterogeneity of individual neighboring neurons. Nevertheless, the observed correlations in both experimental paradigms were rather low, implicating either a high level of variability in the expression profile of individual neurons or the presence of substantial noise in the RNA-Seq data. To further explore the latter possibility, we examined the sequence alignments of the housekeeping gene *Actb* in three neurons from a brain slice (**Figure 4**). These samples differed in their coverage levels for different exons on the 5′-end of mRNA. This observation is consistent with differential degradation of mRNA or synthesis of cDNA, and may contribute to reduction of correlation between RNA-Seq data from different individual cells.

**Table 2:** Pairwise Spearman’s rank correlation coefficient for between all live neurons from brain slice.

ID	Neuron 1	Neuron 2	Neuron 3	4-neuron pool
Neuron 1	_	1183	1244	1323
Neuron 2	0.18	_	1298	1302
Neuron 3	0.30	0.28	_	1715
4-neuron pool	0.29	0.37	0.40	_

*The upper right triangle shows the number of common genes (RKPM > 0 in both samples) used in calculation, whereas the lower left triangle shows the Spearman’s rank correlation coefficients.*

**Table 3:** Pairwise Spearman’s rank correlation coefficient for between all neuronal cells from cell cultures.

ID	Cell 1	Cell 2	Cell 3	Cell 4	Cell 5	Cell 6
Cell 1	_	2555	2824	2063	2376	2696
Cell 2	0.52	_	2599	1914	2213	2508
Cell 3	0.57	0.54	_	2350	2665	3099
Cell 4	0.42	0.35	0.39	_	1955	2155
Cell 5	0.60	0.57	0	61	0.35	_	2595
Cell 6	0.58	0.59	0	61	0.43	0.59	_

*The upper right triangle shows the number of common genes (RKPM > 0 in both samples) used in calculation, whereas the lower left triangle shows the Spearman’s rank correlation coefficients.*

### COMPARISON TO PREVIOUS RNA-Seq AND MICROARRAY STUDIES ON MOUSE TISSUES

To further evaluate the sequencing data with respect to the biology of tissue-specific gene expression, we compared the results to a previous study employing the RNA-Seq technology on mouse tissues (Mortazavi et al., 2008). The previous study examined poly(A)-selected RNA from three mouse tissues (brain, liver, and skeletal muscle), establishing the RPKM as the gold standard for quantifying gene expression levels from RNA-Seq. We found that the Spearman’s rank correlation coefficient between Neuron 1 in the present study and the mouse brain in the Mortazavi et al.’s (2008) study was 0.41. In comparison, the same measure was only 0.18 between Neuron 1 and mouse liver in the Mortazavi et al.’s (2008) study, and only 0.21 between Neuron 1 and mouse muscle in the Mortazavi et al.’s study, respectively. This comparative analysis confirmed that RNA-Seq measurements in our study are more similar to gene expression patterns of brain tissues than other tissues.

We next compared the current single-neuron data to a recent RNA-Seq study on mouse neocortical layers (Belgard et al., 2011). This study sequenced transcriptomes from layers 2 to 6b of different areas (primary and secondary) of the adult mouse somatosensory cortex. The Spearman’s rank correlation coefficients between neuron 1 and their cortical layer 2/3, 4, 5, 6, and 6b neurons are 0.38, 0.33, 0.34, 0.33, and 0.34, respectively. In comparison, the Spearman’s rank correlation coefficients between our cultured Cell 1 and their cortical layer 2/3, 4, 5, 6, and 6b neurons are 0.41, 0.33, 0.36, 0.36, and 0.35, respectively. The slightly increased correlation for cultured neurons as compared to individual neurons may reflect the greater biological variability of neurons from brain slices. The present comparative analysis thus confirmed that data from single-neuron RNA-Seq does indeed recapitulate to expression of genes that are highly expressed in the mouse brain.

To compare our study with previous microarray studies, we further assessed the GNF SymAtlas data set on dozens of mouse tissues, generated on the Affymetrix GNF1M microarray (Su et al., 2004; Lattin et al., 2008). The gene expression values on all tissues were directly taken from the downloaded files. The Spearman’s rank correlation coefficient between Neuron 1 in the present study and cerebral cortex, frontal cortex and hippocampus in the GNF data set were 0.23, 0.22, and 0.21, respectively. In comparison, the correlations of RNA-Seq with microarray data from other mouse tissues were much lower. For example, the correlation with retina, heart, lymph node, pancreas, oocyte, and fertilized egg are 0.15, 0.12, 0.11, 0.09, 0.07, and 0.06, respectively. This comparative analysis thus confirmed that results from single-neuron RNA-Seq were more similar to microarray studies on neuronal tissues than other tissues, but the correlations of measurements were generally very poor.

## CONCLUSION

In this study, we demonstrated the technical feasibility and reproducibility of single-neuron RNA-Seq, as a method that can be applied in standard neuronal cultures or electrophysiology experiments. The present adaptations of the single-neuron RNA-Seq method do not require sophisticated laboratory equipment, making it technically accessible broadly. Furthermore, we compared the reproducibility of RNA-Seq data between different neurons in the same brain slice or different cells in the same neuronal culture. These data suggest that a large portion of the observed differences in gene expression are due to cell-to-cell phenotypic variability rather than noise due to technical issues.

## Conflict of Interest Statement

Gary P. Schroth, Robin Li, and Shujun Luo are employees of Illumina, Inc. The authors declare no other conflict of interests.
