# Erratum: Single-neuron RNA-Seq: technical feasibility and reproducibility

**DOI:** 10.3389/fgene.2013.00023

**Published:** 2013-02-27

**Authors:** Shenfeng Qiu, Shujun Luo, Oleg Evgrafov, Robin Li, Gary P. Schroth, Pat Levitt, James A. Knowles, Kai Wang

**Affiliations:** ^1^Zilkha Neurogenetic Institute, University of Southern CaliforniaLos Angeles, CA, USA; ^2^Department of Cell and Neurobiology, University of Southern CaliforniaLos Angeles, CA, USA; ^3^Illumina, Inc.Hayward, CA, USA; ^4^Department of Psychiatry, University of Southern CaliforniaLos Angeles, CA, USA

To the Editor:

In our manuscript entitled “Single-neuron RNA-Seq: technical feasibility and reproducibility,” published online on July 6th, 2012, we have made a few serious mistakes which need to be corrected:

We referenced several points and quoted statements from a recent study by Okaty et al., but we neither cited the paper nor placed quotation marks on the statements. The full citation should be “Okaty, B. W., Sugino, K., and Nelson, S. B. (2011). Cell type-specific transcriptomics in the brain. *J. Neurosci.* 31, 6939–6943.”

We request to add a citation after “The analysis of each single-cell transcriptome consists of several independent steps” in the first paragraph of Results, as “The analysis of each single-cell transcriptome consists of several independent steps (Okray et al., 2011),” given that these points were previously raised by Okaty et al. We request to add a citation to the first paragraph of Introduction, as “studies of gene expression and function in the brain were restricted to a relatively small number of genes (Luo and Geschwind, 2001; Zhang et al., 2002; Okaty et al., 2011),” given that this points was recently reviewed in the Okaty et al., paper. More importantly, we have made a mistake in quoting sentence from Okray et al., without quotation or citation. We wish to change the following sentence:

Moreover, gene expression may be regulated in opposing directions in different cell types, thereby appearing static in composite data.

To

Moreover, as previously discussed by Okaty et al., “gene expression may be regulated in opposing directions in different cell types, thereby appearing static in composite data (Okaty et al., 2011).”

We apologize to the readers of Frontiers in Genetics and to the authors of the Okaty et al., manuscript, for our failure to correct these mistakes during the review process of our manuscript.

## References

[B1] Luo Z. Geschwind D. H. (2001). Microarray applications in neuroscience. Neurobiol. Dis. 8, 183–193 10.1006/nbdi.2001.0387 11300716

[B2] Okaty B. W. Sugino K. Nelson S. B. (2011). Cell type-specific transcriptomics in the brain. J. Neurosci. 31, 6939–6943 10.1523/JNEUROSCI.0626-11.2011 21562254 PMC3142746

[B3] Zhang W. Wang H. Song S. W. Fuller G. N. (2002). Insulin-likegrowth factor binding protein 2: gene expression microarrays and the hypothesis-generation paradigm. Brain Pathol. 12, 87–94 11770904 10.1111/j.1750-3639.2002.tb00425.x PMC8095777

